# Water-soluble acacetin prodrug confers significant cardioprotection against ischemia/reperfusion injury

**DOI:** 10.1038/srep36435

**Published:** 2016-11-07

**Authors:** Hui Liu, Lei Yang, Hui-Jun Wu, Kui-Hao Chen, Feng Lin, Gang Li, Hai-Ying Sun, Guo-Sheng Xiao, Yan Wang, Gui-Rong Li

**Affiliations:** 1 Department of Medicine, Li Ka Shing Faculty of Medicine, University of Hong Kong, Hong Kong, China; 2 Department of Pharmacology, Tongji Medical College, Huazhong University of Science and Technology, Wuhan, China; 3 Department of Anaesthesiology, Union Hospital, Tongji Medical College, Huazhong University of Science and Technology, Wuhan, China; 4 Shanghai Institute of Pharmaceutical Industry, China National Pharmaceutical Group, Shanghai, China; 5 Xiamen Cardiovascular Hospital, Medical College of Xiamen University, Xiamen, Fujian, China

## Abstract

The morbidity and mortality of patients with ischemic cardiomyopathy resulted from ischemia/reperfusion injury are very high. The present study investigates whether our previously synthesized water-soluble phosphate prodrug of acacetin was cardioprotective against ischemia/reperfusion injury in an *in vivo* rat model. We found that intravenous administration of acacetin prodrug (10 mg/kg) decreased the ventricular arrhythmia score and duration, reduced ventricular fibrillation and infarct size, and improved the impaired heart function induced by myocardial ischemia/reperfusion injury in anesthetized rats. The cardioprotective effects were further confirmed with the parent compound acacetin in an *ex vivo* rat regional ischemia/reperfusion heart model. Molecular mechanism analysis revealed that acacetin prevented the ischemia/reperfusion-induced reduction of the anti-oxidative proteins SOD-2 and thioredoxin, suppressed the release of inflammation cytokines TLR4, IL-6 and TNFα, and decreased myocyte apoptosis induced by ischemia/reperfusion. Our results demonstrate the novel evidence that acacetin prodrug confer significant *in vivo* cardioprotective effect against ischemia/reperfusion injury by preventing the reduction of endogenous anti-oxidants and the release of inflammatory cytokines, thereby inhibiting cardiomyocytes apoptosis, which suggests that the water-soluble acacetin prodrug is likely useful in the future as a new drug candidate for treating patients with acute coronary syndrome.

Ischemic cardiomyopathy resulted from coronary artery disease is the most common cause of mortality worldwide^1^,^2^. Although timely myocardial reperfusion using either thrombolytic therapy or primary percutaneous coronary intervention (PPCI) is the effective therapy in limiting myocardial infarct (MI) size, preserving left-ventricular systolic function and reducing the onset of heart failure, the morbidity and mortality of patients with ischemic cardiomyopathy are significant concerns due to the myocardial reperfusion injury^3^,^4^,^5^. The blood reflow (reperfusion) of the ischemic myocardium induces oxidative stress and a series of intracellular responses, which may trigger life threatening ventricular fibrillation (VF), and may be accompanied with acute inflammatory responses, metabolic disorder, apoptosis and necrosis, and followed by cardiac remodelling and dysfunction^6^,^7^.

Earlier studies on ischemia/reperfusion injury focusing on preconditioning and post-conditioning cardioprotection^3^,^8^ have identified several intracellular molecular signals involved in ischemia/reperfusion injury (e.g. ROS, TNFα, polymorphonuclear leukocytes infiltration, apoptosis signals, etc.) and mobilization of endogenous cardioprotective mechanisms (e.g. adenosine, bradykinin, and opioid peptides, SOD-2, etc.)^7^,^9^. A number of pharmacological agents (e.g. adenosine, beta-blockers, nicorandil, cyclosporine, etc.) have been applied in patients with ischemic heart disorders before thrombolytic therapy or PPCI to reduce reperfusion injury^10^; however, the overall results in clinical trials with the pharmacological recruitment of cardioprotective signaling are not satisfactory, thus no ideal drug is yet available for preventing myocardial reperfusion injury yet^4^. New therapeutic interventions including effective drug therapy are therefore required to improve clinical outcomes^5^,^11^.

In the previous reports, we found that natural flavone acacetin from the Chinese medicinal herb *Tianshanxuelian* inhibits atrial I_Kur_, I_KACh_, and I_to_^12^,^13^,^14^ and prevents the induction of experimental atrial fibrillation in anesthetized canine^12^. We have recently synthesized a water-soluble phosphate prodrug of acacetin, and found that this acacetin prodrug can effectively terminate atrial fibrillation induced by vagal stimulation and burst pacing in beagle dogs after intravenous administration^15^. In the present study, we investigate whether this water-soluble acacetin prodrug is cardioprotective against ischemia/reperfusion injury. Our results demonstrated that intravenous administration of acacetin prodrug was very effective in reducing ventricular arrhythmias and myocardial infarct size induced by ischemia/reperfusion injury in anesthetized rats. Molecular mechanism analysis in *ex vivo* rat hearts revealed that acacetin prevented ischemia/reperfusion reduction of mitochondrial anti-oxidative kinases SOD-2 and thioredoxin as well as reduced inflammatory cytokines TLR4, IL-6 and TNFα, thereby inhibited myocyte apoptosis induced by ischemia/reperfusion.

## Results

### *In vivo* conversion of acacetin prodrug in rats

**Figure 1:**
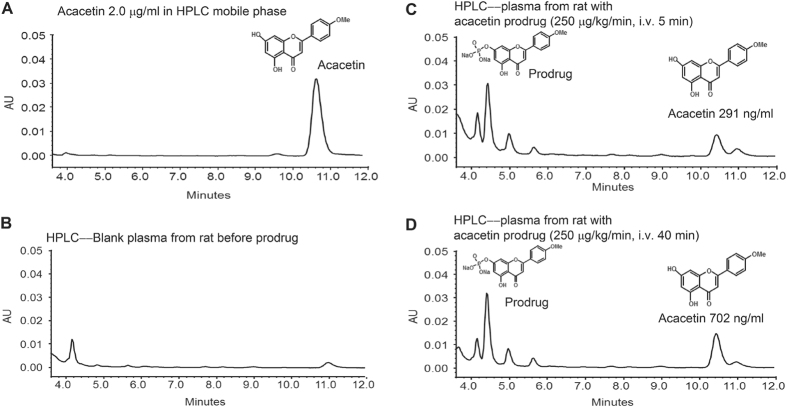
HPLC result for *in vivo* conversion of the water-soluble acacetin prodrug to the parent compound acacetin in a rat after intravenous administration. (**A**) Acacetin (2 μg/mL) in HPLC mobile phase. (**B**) HPLC with blank rat plasma (before administration of acacetin prodrug). (**C**) HPLC with rat plasma at 5 min after intravenous administration of acacetin prodrug (250 μg/kg/min). (**D**) HPLC with rat plasma at 40 min after intravenous administration of acacetin prodrug.

Our recent study has demonstrated that the custom-synthesized acacetin prodrug can be converted to the parent compound acacetin in *in vivo* beagle dogs^15^. The *in vivo* conversion was also confirmed in anesthetized rats. Figure 1 illustrates the representative high-performance liquid chromatography (HPLC) in plasma from an anesthetized rat with intravenous administration (250 μg/kg/min) of acacetin prodrug. The mobile phase containing 2 μg/mL acacetin showed the peak of acacetin on HPLC at 10–11 min. Acacetin was not detected in rat blood plasma before administration of acacetin prodrug (Fig. 1B), and significant plasma level of acacetin was observed at 5 min (Fig. 1C) and 40 min (Fig. 1D) with acacetin prodrug administration. These results indicate that acacetin prodrug is successfully converted *in vivo* into acacetin as reported previously in beagle dogs^15^.

### *In vivo* cardioprotection of acacetin prodrug against ischemia/reperfusion injury in rats

The effect of acacetin prodrug on ventricular arrhythmias induced by ischemia/reperfusion injury was determined in anesthetized rats. Ventricular arrhythmias including premature ventricular beats, ventricular tachycardia, and ventricular fibrillation were induced by ligating the left anterior descending (LAD) artery for 10 min, followed by a 10 min reperfusion as described previously^16^. Acacetin prodrug (5 mg, 10 mg and 20 mg/kg) or equivolume vehicle (5% glucose solution) was intravenously administered for 5 min before the artery ligation.

**Figure 2:**
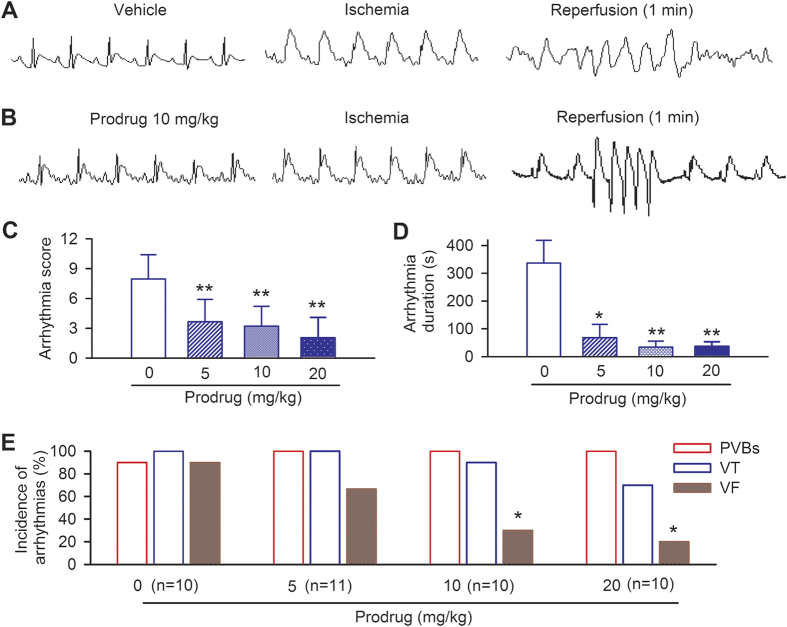
Anti-arrhythmic effect of prodrug in anesthetized rats with ischemia/reperfusion. (**A**) ECG in a rat treated with vehicle, showing ventricular fibrillation (VF) after reperfusion for 1 min. (**B**) ECG in a rat treated with bolus acacetin prodrug (10 mg/kg i.v. for 5 min) before ischemia, showing a brief ventricular tachycardia (VT) followed by normal rhythm after reperfusion. (**C**) Reduction of the ventricular arrhythmia score by acacetin prodrug. (**D**) Decrease of ventricular arrhythmia duration by acacetin prodrug. (**E**) Reduction of ventricular fibrillation (but not PVBs, premature ventricular beats) by acacetin prodrug in a concentration-dependent manner (n = 10–11 experiments, *P < 0.05, **P < 0.01 vs. vehicle).

Ventricular fibrillation was frequently observed during the early reperfusion in control animals treated with intravenous vehicle (Fig. 2A), but not in animals treated intravenously with acacetin prodrug (Fig. 2B). Acacetin prodrug decreased the arrhythmia score (Fig. 2C) and arrhythmia duration (Fig. 2D) in a dose-dependent manner (n = 10–11, *P* < 0.05 or *P* < 0.01 vs. vehicle). Importantly, incidence of ventricular fibrillation was reduced by 26%, 67%, and 78% with bolus acacetin prodrug of 5, 10 and 20 mg/kg (Fig. 2E, *P* < 0.05 for 10 mg/kg or 20 mg/kg vs. vehicle).

**Figure 3:**
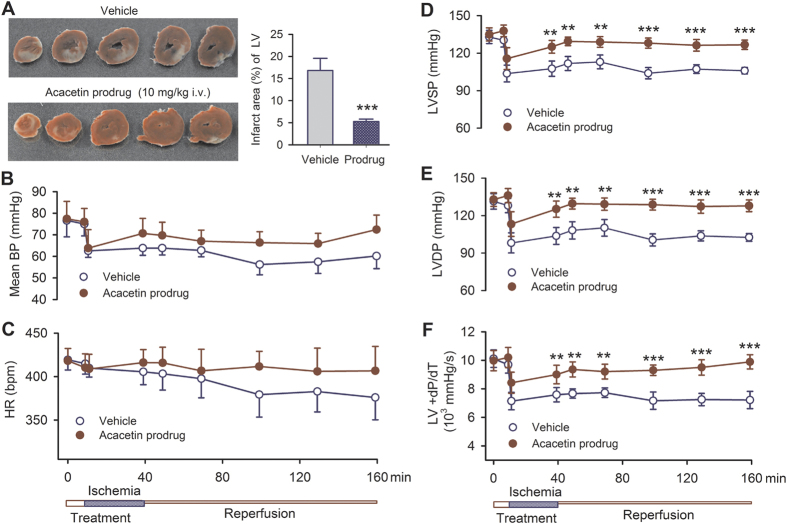
Intravenous administration of prodrug improves the impaired heart rate and ventricular contractile function induced by ischemia/reperfusion injury in anesthetized rats. (**A**) Left ventricular slices with the TTC-staining, showing a reduced infarct area in animal treated with acacetin prodrug (10 mg/kg intravenous administration). (**B**) Acacetin prodrug antagonizes the reduction of mean femoral artery blood pressure by ischemia/reperfusion injury. (**C**) Acacetin prodrug slightly antagonizes the heart rate reduction by ischemia/reperfusion injury. (**D**) Acacetin prodrug improves the impaired systolic pressure of left ventricle (LVSP). (**E**) Acacetin prodrug improves the impaired developed pressure of left ventricle (LVDP). (**F**) Acacetin prodrug antagonizes the reduced left ventricular +dP/dT (n = 8 experiments, **P < 0.01, ***P < 0.001 vs. vehicle).

The anti-infarct effect of acacetin prodrug was determined in anesthetized rats with 30 min LAD artery ligation, followed by 120 min reperfusion. Acacetin prodrug was administered by intravenous injection (10 mg/kg) before 10 min of the LAD artery ligation. Ventricular infarct size was remarkably reduced by 68.7% in rats treated with acacetin prodrug (Fig. 3A, n = 8 for each group, *P* < 0.001 vs. vehicle).

Moreover, the prodrug prevented the reduction of the mean femoral artery blood pressure (Fig. 3B) and the heart rate (Fig. 3C). It significantly improved the impaired ventricular contractile function, i.e. left ventricular systolic pressure (Fig. 3D, *P* < 0.01 or *P* < 0.001 vs. vehicle), developed pressure (Fig. 3E, *P* < 0.01 or *P* < 0.001 vs. vehicle), and +dP/dT (Fig. 3F, *P* < 0.01 or *P* < 0.001 vs. vehicle), induced by ischemia/reperfusion injury. These results show that acacetin prodrug is very effective in inhibiting ventricular arrhythmias, reducing infarct size, and preserving heart function in rat *in vivo* ischemia/reperfusion model. These results also indicate that acacetin prodrug is converted to the parent compound acacetin and exerts significant cardioprotective action against ischemia/reperfusion injury.

### *Ex vivo* cardioprotection of acacetin against ischemia/reperfusion injury

**Figure 4:**
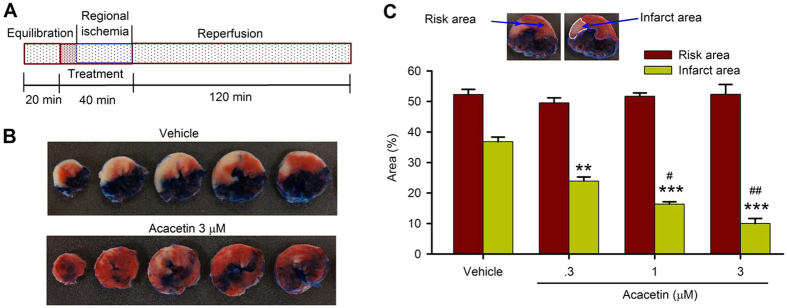
Effects of acacetin on myocardial injury by ischemia/reperfusion in *ex vivo* rat hearts. (**A**) The scheme of ischemia/reperfusion protocol in perfused *ex vivo* rat hearts with 30 min ischemia, followed by 2 h reperfusion. (**B**) Left ventricular slices of the heart with Evans blue/TCC-staining in a vehicle control heart and a heart treated with 3 μM acacetin. (**C**) Mean percentage of risk area and infarct area of left ventricle with ischemia/reperfusion injury, showing reduction of infarct area in hearts treated with 0.3, 1 or 3 μM in a concentration-dependent manner (n = 10–12 experiments, **P < 0.01, ***P < 0.001 vs. vehicle; ^#^*P* < 0.05, ^##^*P* < 0.01 vs. 0.3 μM acacetin).

**Figure 5:**
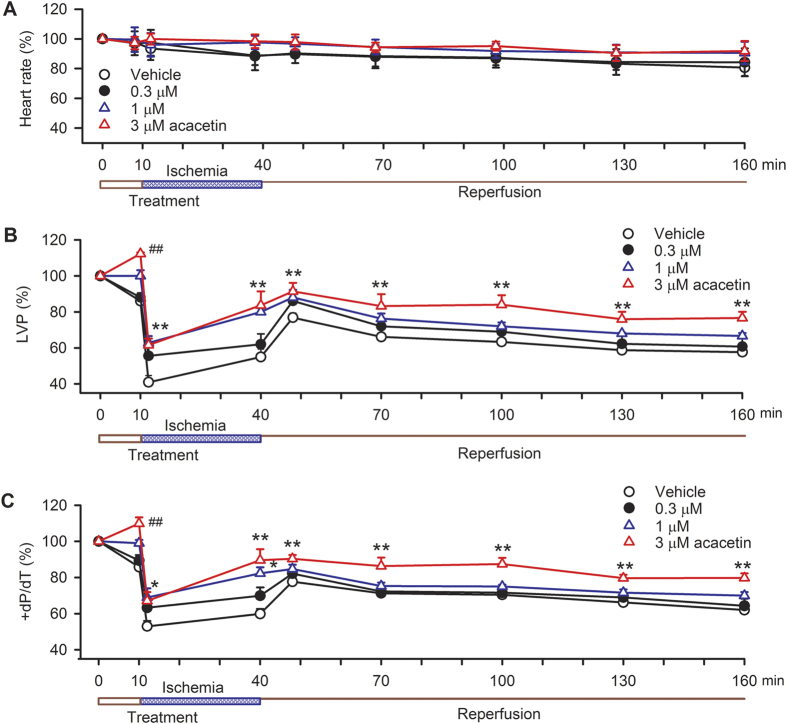
Effects of acacetin on heart rate and ventricular pressure in *ex vivo* rat hearts subjected to ischemia/reperfusion. (**A**) Percentage values of heart rate during control, ischemia and reperfusion in isolated rat hearts treated with vehicle, 0.3, 1 or 3 μM acacetin. (**B**) Percentage values of left ventricular pressure (LVP) during control, ischemia and reperfusion in isolated rat hearts treated with vehicle, 0.3, 1 or 3 μM acacetin (n = 9–10 for each treatment, ^##^P < 0.01 vs control; *P < 0.05, **P < 0.01 vs. vehicle). (**C**) Percentage values of +dP/dT of left ventricular pressure during control, ischemia and reperfusion in isolated rat hearts treated with vehicle, 0.3, 1 or 3 μM acacetin (n = 9–10 for each treatment, ^##^P < 0.01 vs control; *P < 0.05, **P < 0.01 vs. vehicle).

To determine the potential molecular mechanisms underlying cardioprotection of acacetin prodrug, the parent compound acacetin was used to test the effect on myocardial ischemia/reperfusion injury in isolated rat hearts. The perfused hearts were subjected to LAD artery ligation for 30 min followed by 120 min reperfusion. The hearts were treated with different concentrations of acacetin before (10 min) and during the regional ischemia (Fig. 4A). The infarct area assessed by Evans blue/TTC staining in myocardial slices showed that a sizeable infarct area was present in the heart treated with vehicle, and the infarct size was remarkably reduced in the heart treated with 3 μM acacetin (Fig. 4B). The mean values (Fig. 4C) of the infarct area induced by ischemia/reperfusion injury was decreased by 36%, 54% and 73% respectively with 0.3, 1 and 3 μM acacetin (n = 10–11, *P* < 0.05 or *P* < 0.001 vs. vehicle). Significant reduction of the infarct size was observed at concentration as low as 0.3 μM acacetin. In addition, the heart function was also evaluated in isolated rat hearts subjected to ischemia/reperfusion injury (Fig. 5). LVP and +dP/dT was significantly reduced by ischemia/reperfusion. Acacetin at 3 μM acacetin significantly improved the impaired heart function by ischemia/reperfusion (*P* < 0.05 or *P* < 0.001 vs. vehicle).

It is well documented that myocardial ischemia/reperfusion injury induces intracellular oxidative stress, triggers inflammation, and initiates apoptosis signals to mediate the subsequent myocardial death^6^,^7^,^17^. Therefore, we determined whether the remarkable cardioprotection of acacetin involves anti-oxidation, anti-inflammation or anti-apoptosis.

### Molecular mechanisms of cardioprotection of acacetin against myocardial ischemia/reperfusion injury

**Figure 6:**
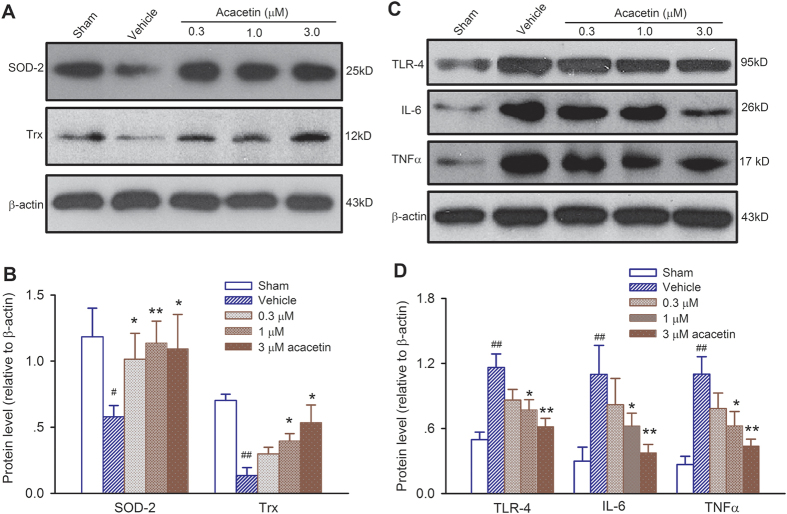
Effects of acacetin on endogenous anti-oxidant and inflammation response in *ex vivo* rat hearts with ischemia/reperfusion injury. (**A**) Western blots of superoxide dismutase 2 (SOD-2) and thioredoxin (Trx). (**B**) Mean relative levels of SOD-2 and Trx. (**C**) Western blots of Toll like receptor-4 (TLR-4), interleukin-6 (IL-6), and tumour necrosis factor alpha (TNFα) in rat left ventricles with ischemia/reperfusion injury in the absence and the presence of 0.3, 1 and 3 μM acacetin. (**D**) Mean relative levels of TLR-4, IL-6 and TNFα, showing significant reduction of TLR-4, IL-6 and TNFα in hearts treated with acacetin (0.3, 1 and 3 μM). n = 4 experiments, ^#^*P* < 0.05, ^##^*P* < 0.01 vs. Sham; *P < 0.05, **P < 0.01 vs. vehicle.

Previous studies demonstrated that the anti-oxidative proteins SOD (superoxide dismutase) and/or thioredoxin are decreased in myocardial ischemia/reperfusion injury or other oxidation stress^18^,^19^. Similarly, in isolated hearts with ischemia/reperfusion injury, we also found (Fig. 6A,B) that SOD-2 and thioredoxin were remarkably reduced in the left ventricle of rat hearts subjected to 30 min ischemia followed by 2 h reperfusion (n = 4, P < 0.01 vs. sham group). Interestingly, acacetin treatment prevented SOD-2 and thioredoxin reduction induced by ischemia/reperfusion injury (n = 4, *P* < 0.05 or *P* < 0.01 vs. vehicle), indicating that cardioprotective effects of acacetin involve the retention of endogenous anti-oxidants.

The release of inflammation mediators (TLR-4, IL-6, TNFα, etc.) has been found to play an important role in mediating and exacerbating myocardial ischemia/reperfusion injury^7^,^17^. Figure 6C,D show that TLR-4, IL-6 and TNFα were remarkably increased in *ex vivo* rat hearts subjected to ischemia/reperfusion injury (n = 4, *P* < 0.01 vs. sham group), and acacetin decreased the inflammatory responses in a concentration-dependent manner (n = 4 for each group, *P* < 0.05 or *P* < 0.01 vs. vehicle).

**Figure 7:**
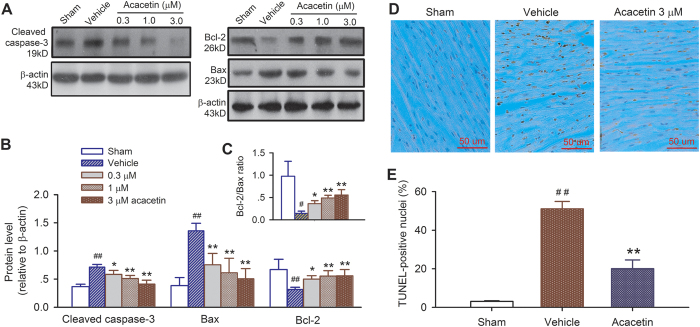
Effects of acacetin on myocardial apoptosis induced by ischemia/reperfusion in *ex vivo* rat hearts. (**A**) Western blots for expression of the pro-apoptotic proteins cleaved caspase-3, Bax and the anti-apoptotic protein Bcl-2 in the absence and the presence of 0.3, 1 and 3 μM acacetin. (**B**) Mean relative protein levels. (**C**) Ratio of Bcl-2/Bax. n = 4 experiments, ^#^*P* < 0.05, ^##^*P* < 0.01 vs. sham; *P < 0.05, **P < 0.01 vs. vehicle. (**D**) TUNEL-staining indicates significant myocyte apoptosis with dark-brown nuclei; acacetin decreases apoptosis. (**E**) Mean percentage of nuclei with TUNEL-staining in hearts treated with vehicle or 3 μM acacetin (n = 5 experiments, ^##^*P* < 0.01, vs. sham; **P < 0.01 vs. vehicle).

The potential involvement of molecular mechanism of anti-apoptosis by acacetin was further determined in *ex vivo* rat hearts subjected to ischemia/reperfusion insult. The pro-apoptotic proteins cleaved caspase-3 and Bax (Bcl-2-associated X), as well as the anti-apoptotic protein Bcl-2 (B-cell lymphoma 2) were examined in hearts treated with vehicle, 0.3, 1, or 3 μM acacetin. Figure 7A,B show that ischemia/reperfusion increased the pro-apoptotic cleaved caspase-3 and Bax and decreased the anti-apoptotic Bcl-2 (n = 4, *P* < 0.01 vs. sham). Acacetin significantly antagonized the decrease of anti-apoptotic molecule (Bcl-2) and the increase of pro-apoptotic molecules (Bax and caspase-3) induced by ischemia/reperfusion injury (n = 4, *P* < 0.05 or *P* < 0.01 vs. vehicle). The reduced Bcl-2/Bax ratio was reversed by acacetin treatment in a concentration-dependent manner (Fig. 7C, n = 4, *P* < 0.05 or *P* < 0.01 vs. vehicle). These results indicate that acacetin also has a secondary anti-apoptosis effect in addition to its anti-oxidation and anti-inflammation cardioprotective properties.

The TUNEL-staining for detecting apoptotic cells in left ventricular slices (Fig. 7D) shows that the increase of TUNEL-positive nuclei cells by ischemia/reperfusion injury was reduced in hearts treated with 3 μM acacetin (Fig. 7E). The TUNEL-positive nuclei was 51.1 ± 3.8% in vehicle control hearts, and was reduced to 20.2 ± 4.1% in acacetin-treated hearts (n = 5, *P* < 0.01 vs. vehicle), indicating that acacetin inhibits myocytes apoptosis thereby decreases myocardial infarct size in rat hearts subjected to ischemia/reperfusion injury. The histological damage induced by ischemia/reperfusion was also attenuated in hearts treated with 3 μM acacetin (Fig. S1).

## Discussion

In the present study, we have demonstrated for the first time that the water-soluble prodrug of the natural flavone acacetin is converted into the parent compound acacetin and exerts a remarkable cardioprotective effect in anesthetized rats subjected to ischemia/reperfusion injury: reducing ventricular arrhythmia score and duration, ventricular fibrillation and myocardial infarct size and improving heart function. Molecular mechanisms of acacetin for cardioprotection determined in *ex vivo* rat hearts with ischemia/reperfusion injury reveals that acacetin prevents ischemia/reperfusion-induced reduction of anti-oxidants, decreases inflammation responses, and inhibits myocardial apoptosis.

Acacetin is a small molecule flavone (5,7-dihydroxy-4′-methoxyflavone) universally present in various plants and responsible for many of the colours in nature^20^. In addition to our previous reports of atrial-selective ion channels (I_Kur_, I_KACh_, and I_to_) blockade and anti-atrial fibrillation effect^12^,^13^,^14^, acacetin has been reported to have anti-cancer properties^21^, to reduce the airway hyper-responsiveness with dietary administration^22^, and to alleviate telomeric position effect in human cells^23^. The present study demonstrates that acacetin has the novel therapeutic cardioprotection against ischemia/reperfusion injury.

It has been well documented that myocardial ischemia/reperfusion (oxidative stress) stimulates the generation of reactive oxygen species (ROS) involved in myocardial injury^3^. These reactive oxygen species injure membrane lipids, protein, carbohydrates and DNA, and cause widespread inflammation associated with tissue damage^24^,^25^, which is an active process of cardiomyocyte apoptosis and/or necrosis^26^,^27^. Therefore, scavenging oxidative species or enhancing endogenous antioxidants could prevent the myocardial injury induced by oxidation. Cardioprotection relies on a complex signal transduction of triggers, mediators and effectors, which vary between different species^28^,^29^. It is generally believed that the main endogenous antioxidant is superoxide dismutase (SOD), which catalyses O_2_ reduction to produce H_2_O_2_. In patients and animals with myocardial ischemia/reperfusion, tumour necrosis factor-alpha (TNFα) and interleukin-6 (IL-6) were increased with other inflammatory cytokines^30^.

It should be noted that TNFα has an ambivalent role in myocardial ischemia/reperfusion injury. Both local ischemic conditioning and remote ischemic postconditioning are related to the decrease of the endogenous TNFα induced by myocardial ischemia/reperfusion in rodents^31^. On the other hand, evidence that ischemic preconditioning protection is lost in TNFα-KO mice and exogenous TNFα mimics the ischemia-preconditioning cardioprotection when administered prior to ischemia suggest that TNFα acts as a trigger of preconditioning^32^,^33^. Exogenous TNFα has been found to have biphasic effects: it is cardioprotective at low concentrations and acts as a signaling molecule, but it induces irreversible cell damage and increases infarct size at higher concentrations^32^,^34^,^35^.

**Figure 8:**
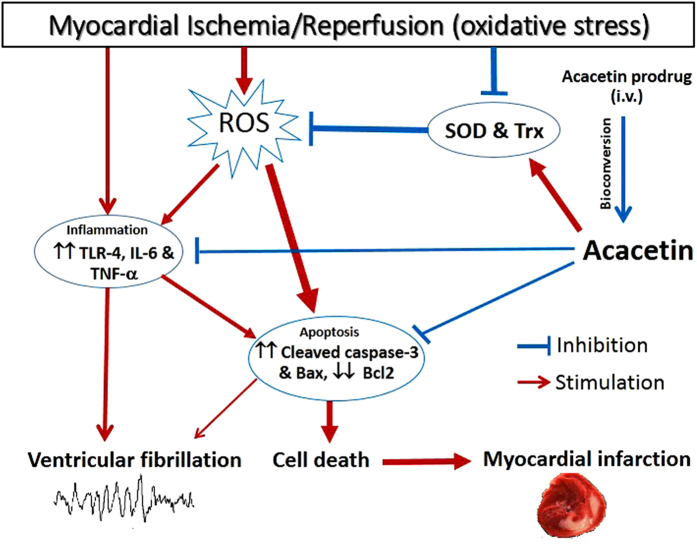
Schematic illustration shows the main cascade of molecules in myocardial ischemia/reperfusion injury. Myocardial ischemia/reperfusion (oxidative stress) triggers ROS production and inflammation response (release of pro-inflammatory cytokines TLR-4, IL-6, TNFα, which induce ventricular arrhythmias) and decreases the endogenous anti-oxidants SOD and thioredoxin (Trx). Over-production of ROS and inflammation cytokines would initiate cardiomyocyte apoptosis cell death and cause myocardial infarct. Intravenous application of acacetin prodrug (bioconversion to acacetin) prevents the ischemia/reperfusion-induced reduction of the endogenous anti-oxidants and also reduces inflammation response, and thereby inhibits ventricular arrhythmias and myocardial apoptosis and significantly reduces infarct size.

It is well recognized that the overall preservation of cardiac structure and function is critical for cardioprotection against ischemia/reperfusion injury by inhibiting oxidative stress and inflammation and therefore decreasing apoptosis^17^,^36^,^37^. Earlier studies reported that preserving anti-oxidant systems using recombinant SOD^38^,^39^ or thioredoxin^40^,^41^,^42^ had significant cardioprotection against ischemia/reperfusion injury. Consistent with the previous reports^3^,^7^, the present study also showed that the endogenous antioxidant proteins SOD-2 and thioredoxin, and the anti-apoptotic Bcl-2 were remarkably reduced in rat hearts subjected to ischemia/reperfusion injury, while pro-inflammatory factors Toll-like receptor 4 (TLR-4)^43^, IL-6 and TNFα, the pro-apoptotic molecules cleaved caspase-3 and Bax were significantly increased by ischemia/reperfusion injury. It is interesting to note that acacetin treatment remarkably prevented the ischemia/reperfusion–induced reduction of SOD-2 and Bcl-2, preserved their activities, and thereby inhibited inflammation mediators (TLR-4, IL-6, and TNFα) and cardiomyocyte apoptosis and decreased the myocardial infarct size (Fig. 8). The preservation of the anti-oxidant systems (SOD and thioredoxin) may be the key effect of acacetin for cardioprotection against ischemia/reperfusion injury.

It is generally recognized that cardiomyocytes suffer irreversible injury primarily during ischemia/reperfusion, the window time is important for clinical use of cardioprotective drugs to minimize additional cardiomyocyte loss during reperfusion. A large number of studies have recommended introducing protective drugs at the very beginning of reperfusion to reduce infarct size^44^. It is believed that the protective effect will be enhanced if cardioprotection is initiated before or during ischemia or as soon as possible after reperfusion, especially with longer duration of ischemia^4^,^5^. Although a number of clinical studies on cardioprotection were conducted with promising pharmaceutical agents, including the endogenous cytoprotective molecule adenosine, the mitochondrial permeability transition pore formation blocker cyclosporine A^45^, and the vasodilators nicorandil and nitrates^46^, beta-blockers^4^,^5^,^11^, the long term results were not satisfactory. Therefore, more effort is required to develop new cardioprotective strategies to improve the outcomes of acute myocardial infarction^4^,^5^,^11^. The present study demonstrated that acacetin prodrug has significant cardioprotection against ischemia/reperfusion injury by reducing ventricular fibrillation and infarct size and preserving ventricular contractile function.

Although the present study indicates that acacetin prodrug is likely a promising drug candidate to rescue the myocardial injury on reperfusion, the limitation is that all the results were obtained in rodents. Further experimental study is required in large animals (e.g. swine and/or canine) to gain the preclinical data which can be referred for clinical trials in the near future.

## Conclusion

The present study demonstrates for the first time that acacetin prodrug confers significant cardioprotective effect against ischemia/reperfusion injury after converting into the parent compound acacetin by preventing ischemia/reperfusion-induced decrease of anti-oxidants, thereby reducing inflammatory response and inhibiting myocardial apoptosis and infarct size, which suggests that acacetin prodrug may improve the long term outcomes of patients undergoing thrombolytic therapy or PPCI.

## Methods and Materials

### Animal experiments

Sprague Dawley (SD) rats were employed in the present study following the Guide for the Care and Use of Laboratory Animals published by the US National Institutes of Health (NIH Publication No. 85-23, revised 1996), and the experimental procedure was approved by the Committee on the Use of Live Animals in Teaching and Research of the University of Hong Kong.

### Blood sample collection for determining prodrug conversion in anesthetized rats with HPLC analysis

Adult male SD rats (250–300 g) was anesthetized with pentobarbital (50 mg/kg, i.p.), and the jugular vein and femoral vein were cannulated for drug administration and blood sample collection respectively. The water-soluble acacetin prodrug (Fig. S2) with purity of 99% (Fig. S3) was continuously administered for 40 min (250 μg/kg/min), and blood sample (~0.2 mL) was collected in heparinized tubes at 0 (pre-dose) and 5, 10, and 40 min during drug administration for HPLC analysis of acacetin prodrug conversion.

The simple liquid-liquid extraction with methanol was used for sample preparation. Pentamethylquercetin was selected as internal standard. The analysis was carried out on a Waters HPLC system (Milford, MA) equipped with an Alltech column (C18, 250 mm × 4.6 mm i.d., 5 μm, Grace, Deerfield, IL). The mobile phase consisted of methanol-water-phosphoric acid (70:30:0.15), and the flow rate was 1.0 mL/min. UV absorption was monitored at 260 nm.

### Myocardial ischemia/reperfusion model in anesthetized rats

Ischemia-reperfusion model in anesthetized rats was established as described previously^16^. Adult male SD rats were anesthetized with pentobarbital (50 mg/kg, i.p.) and, supplemented during the experiment when needed. The animals were intubated and ventilated with room air. Body temperature was maintained at 37 °C with a temperature control system. The jugular vein was cannulated for drug administration and a PE-50 catheter was introduced into the left ventricle through right carotid artery to measure ventricular contractile function (e.g. left ventricular pressure). Femoral artery was cannulated to measure blood pressure. Lead II ECG was monitored during the experiment with a data acquisition system (RM6240, Chengdu Instrument Ltd, Chengdu, China). Regional ischemia was achieved by ligating LAD artery using a 5–0 silk suture with a section of silica gel tubing. Myocardial ischemia was confirmed by regional cyanosis and ST-segment elevation.

After 10-min stabilization, acacetin prodrug (5 mg, 10 mg and 20 mg/kg) or equivolume vehicle (5% glucose solution) was intravenously administered for 5 min before LAD artery was ligated for 10 min, followed by a 10-min reperfusion. Cardiac injury was characterized by a high incidence of ventricular arrhythmias during reperfusion in this model. Definitions of arrhythmias were based on the criteria of the Lambeth Conventions, including premature ventricular contractions (PVCs), ventricular tachycardia (VT), and ventricular fibrillation (VF)^47^. An arrhythmia score was calculated with scoring system as previously described: [(Log_10_PVCs) + 2 (Log_10_ episodes of VT) + 2 (Log_10_ episodes of VF) + (Log_10_ total duration of VF)]^48^,^49^.

To evaluate the ischemia/reperfusion-induced myocardial infarct size, rats were anesthetized and subjected to 30 min LAD artery ligation and followed by 120 min reperfusion. Acacetin prodrug (10 mg/kg, i.v.) was continuously administered for 10 min before LAD artery ligation and during 30 min ischemia. Lead II ECG, blood pressure, and left ventricular pressure were monitored and recorded on an IBM compatible PC computer using a data acquisition system (RM6240, Chengdu Instrument Ltd, Chengdu, China) for offline analysis.

### *Ex vivo* rat myocardial ischemia/reperfusion model

Adult male SD rats were anesthetized, their hearts were isolated and retrogradely perfused on a Langendorff heart perfusion apparatus at 37 °C with modified Krebs-Henseleit solution (in mM): NaCl 118.5, KCl 4.7, KH_2_PO_4_ 1.18, MgSO_4_ 1.18, NaHCO_3_ 25, glucose 11.1, CaCl_2_ 2.5) gassed with 95% O_2_ and 5% CO_2_. Polyethylene tubing with a cling film balloon tip filled with water and connected to a pressure transducer was inserted into the left ventricle through a small incision in the left atrium to measure left ventricular pressure. ECG was recorded with two electrodes placed on heart surface and the physiological signals were saved on an IBM compatible PC computer using the data acquisition system (Chengdu Instrument Ltd).

Regional ischemia was achieved by ligating LAD artery using a 5–0 silk suture with a section of silica gel tubing. After 20-min equilibration, the hearts were treated by vehicle (DMSO) or different concentrations of acacetin (0.3, 1 or 3 μM) for 10 min, and during LAD artery ligation (30-min), followed by 2 hr reperfusion. Myocardial ischemia was confirmed by a decrease in left ventricular developed pressure (LVDP) and specific ST-segment elevation on ECG. The hearts were used to perform histological and molecular analysis at end of 2 hr reperfusion.

Data were discarded if one of the following criteria appeared: 1) LVDP was below 80 mmHg during equilibration, 2) lack of evidence of successful LAD occlusion, and 3) hearts with irreversible arrhythmia during equilibration or after ischemia.

### Myocardial infarct size assessment

At end of reperfusion, LAD artery was re-occluded at the same location. 0.5% solution of Evans blue dye was infused via aorta to differentiate the area at risk from the non-ischemic zone. The heart was then rapidly excised and frozen at −20 °C for 2–3 hr, and cut into 2-mm transverse slices from apex to base. The slices were incubated in 1% triphenyltetrazolium chloride (TTC) in sodium phosphate buffer (pH 7.4) for 20 min at 37 °C and fixed in 10% formalin.

After Evans blue/TTC staining, myocardial ischemic risk area was identified as the myocardial tissue without blue staining and presented as a percentage of the total left ventricular area. Viable tissue in the risk area (deep red) was stained by TTC, while infarcted area was clearly visible as TTC-negative (pale) and expressed as a percentage of total myocardial risk area. Images of the heart slices were taken and size of risk area and infarct area was measured by Image J software.

### Histopathological analysis

After the reperfusion, the heart was fixed with 10% buffered formalin, embedded in paraffin, and then sectioned into 5 μm slices. The tissue slices were stained with haematoxylin-eosin, and analysed by light microscopy.

### Terminal deoxynucleotidyl transferase–mediated dUTP nick end labeling (TUNEL) assay

TUNEL staining of myocardial slices was performed to detect myocyte apoptosis in myocardium using an *in situ* cell death detection kit, POD (Roche Applied Sciences, Mannheim, Germany) following the manufacturer’s instruction. Samples were counterstained with haematoxylin prior to analysis by light microscope. Quantitative analysis of TUNEL-positive cells was performed in 10 random fields at 100× magnification on three different sections from the peri-infarction zone in each animal as reported previously^50^,^51^. Apoptotic myocytes were expressed as percentage of total cells in the field.

### Western blotting analysis

The related proteins were determined with Western blotting analysis as described previously^52^. Myocardial tissue samples from the ischemic zone were homogenized with an ice cold modified RIPA buffer. Protein concentration was determined using a Bio-Rad protein assay (Bio-Rad Laboratories, Hercules, CA). Equal concentrations of proteins were mixed with SDS sample buffer and denatured at 95 °C for 5 min. The samples were resolved with 8% SDS–page gels which were then transferred onto nitrocellulose membranes. The membranes were blocked with 5% fat-free milk in 0.1% Tris-buffered saline with Tween (TTBS) for 2 hours and then probed with primary antibodies: anti-SOD-2, thiredoxin, TLR-4, TNFα, caspase-3, Bax and Bcl-2 antibodies (Santa Cruz Biotechnology, USA); anti-IL-6 antibody (Abcam, UK) at 4 °C overnight. After being washed for three times with TTBS, the membranes were incubated with secondary antibodies (Santa Cruz Biotechnology) in TTBS at room temperature for 2 hours. Membranes were washed again with TTBS three times and then visualized on X-ray films using a chemo-luminescence detection system (ECL, GE Healthcare). The housekeeping protein β-actin (Santa Cruz Biotechnology) was used to protein loading control. The relative band intensities were measured by image analysis software Gel-Pro Analyser.

### Statistical analysis

Group data are expressed as mean ± S.E.M. The incidence of arrhythmias was analyzed using the Fisher exact test. Arrhythmia scores were analyzed using one-way ANOVA followed by a Tukey *post hoc* test. Quantitative data were analyzed using Student’s t-test for paired or unpaired observations to evaluate significant differences between two group means, and ANOVA for multiple groups. A value of *P* < 0.05 was taken to indicate statistical significance.

## Additional Information

**How to cite this article**: Liu, H. *et al.* Water-soluble acacetin prodrug confers significant cardioprotection against ischemia/reperfusion injury. *Sci. Rep.* **6**, 36435; doi: 10.1038/srep36435 (2016).

**Publisher’s note:** Springer Nature remains neutral with regard to jurisdictional claims in published maps and institutional affiliations.

## Supplementary Material

Supplementary Information
