# Supplementary material for: Water-soluble acacetin prodrug confers significant cardioprotection against ischemia/reperfusion injury
Source: Sci Rep. 2016 Nov 7;6:36435. doi: 10.1038/srep36435 (PMC5098248; doi:10.1038/srep36435)
Supplement: Supplementary Information [file srep36435-s1.pdf]

Water-soluble acacetin prodrug confers significant cardioprotection against ischemia/reperfusion injury

Hui Liu, Lei Yang, Hui-Jun Wu, Kui-Hao Chen, Feng Lin, Gang Li, Hai-Ying Sun, Guo-Sheng Xiao, Yan Wang, Gui-Rong Li

Supplemental material

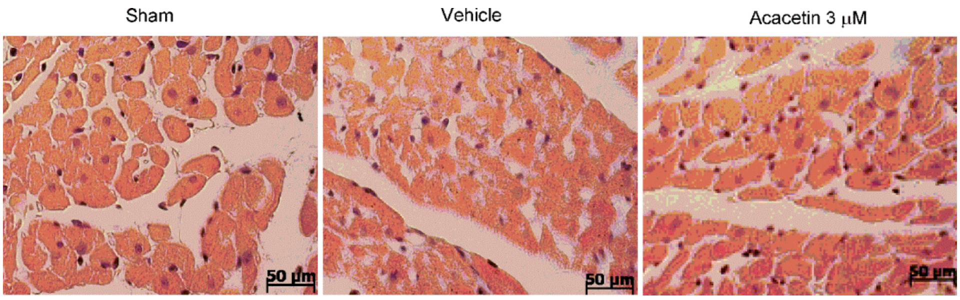

Figure S1. HE staining of ventricular slices from *ex vivo* rat hearts.

Sham: Sham rat heart tissue without ischemia/reperfusion.

Vehicle: Vehicle-treated rat heart tissue with ischemia/reperfusion.

Acacetin 3 μM: Rat heart treated using 3 μM acacetin with ischemia/reperfusion. Significant histological alteration was observed in vehicle-treated heart. Tissue damage was clearly attenuated in acacetin-treated heart.

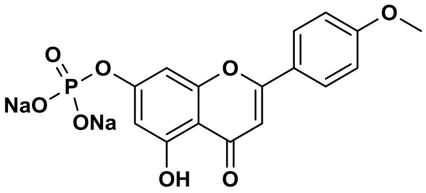

Figure S2. Structure of prodrug

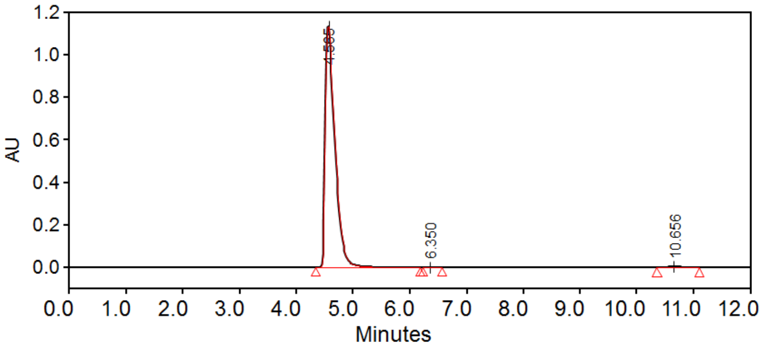

UV HPLC 1 result

Peak #	Retention Time	% Area
1	4.57	99.04
2	6.35	0.05
3	10.66	0.91
Totals		100.00

Figure S3. HPLC graph of prodrug showing the purity of 99.04%.
